# A Practice‐Based, Clinical Pharmacokinetic Study to Inform Levetiracetam Dosing in Critically Ill Patients Undergoing Continuous Venovenous Hemofiltration (PADRE‐01)

**DOI:** 10.1111/cts.12782

**Published:** 2020-04-03

**Authors:** Shamir N. Kalaria, Michael Armahizer, Paul McCarthy, Neeraj Badjatia, Jogarao V. Gobburu, Mathangi Gopalakrishnan

**Affiliations:** ^1^ Center for Translational Medicine University of Maryland School of Pharmacy Baltimore Maryland USA; ^2^ Department of Pharmacy Services University of Maryland Medical Center Baltimore Maryland USA; ^3^ Department of Cardiovascular and Thoracic Surgery Division of Critical Care West Virginia University School of Medicine Morgantown West Virginia USA; ^4^ Department of Neurology University of Maryland School of Medicine Baltimore Maryland USA

## Abstract

Limited data exist on the effect of continuous renal replacement therapy (CRRT) methods on anti‐epileptic drug pharmacokinetics (PK). This prospective practice‐based PK study aims to assess the impact of continuous venovenous hemofiltration (CVVH), a modality of CRRT, on levetiracetam PK in critically ill patients and to derive individualized dosing recommendations. Eleven patients receiving oral or intravenous levetiracetam and CVVH in various intensive care units at a large academic medical center were enrolled to investigate the need for dosing adjustments. Prefilter, postfilter, and ultrafiltrate samples were obtained before dosing, after the completion of the infusion or 1‐hour postoral dose, and up to 6 additional time points postinfusion or postoral administration. Patient‐specific blood and ultrafiltrate flow rates and laboratory values were also collected at the time of sampling. The average sieving coefficient (SC) for levetiracetam was 0.89 ± 0.1, indicating high filter efficiency. Six of the 11 patients experienced concentrations outside the reported therapeutic range (12–46 mg/L). The average volume of distribution was 0.73 L/kg. CVVH clearance contributes a major fraction of the total levetiracetam clearance (36–73%) in neurocritically ill patients. The average bias and precision of the estimated vs. observed total clearance value was ~ 10.6% and 21.5%. Major dose determinants were identified to be SC and effluent flow rate. Patients with higher ultrafiltrate rates will have increased drug clearance and, therefore, will require higher doses in order to match exposures seen in patients with normal renal function.

**Study Highlights**

**WHAT IS THE CURRENT KNOWLEDGE ON THE TOPIC?**

☑ Currently, there are no guidelines established by the US Food and Drug Administration regarding the use of drugs with continuous renal replacement therapy (CRRT). CRRT pharmacokinetic (PK) studies are also not required for new drug approvals.

**WHAT QUESTION DID THIS STUDY ADDRESS?**

☑ The objectives of this prospective, “real‐world,” PK study are to characterize the PK of levetiracetam in critically ill patients undergoing continuous venovenous hemofiltration (CVVH) and to derive CVVH‐specific dosing recommendations to achieve a target exposure range found in patients with normal renal function.

**WHAT DOES THIS STUDY ADD TO OUR KNOWLEDGE?**

☑ This proof of principle study confirms that PK data collected in course of clinical care in patients undergoing CVVH can be leveraged to optimize levetiracetam dosing recommendations. Effluent flow rate and sieving coefficient (SC) were found to be the major determinants for drug clearance and dosing. Although the estimated SC was similar to the fraction unbound for levetiracetam, no significant correlation was found between transmembrane/filter pressure and SC.

**HOW MIGHT THIS CHANGE CLINICAL PHARMACOLOGY OR TRANSLATIONAL SCIENCE?**

☑ In order to avoid overdosing that could lead to potential toxicity and underdosing that could compromise efficacy, a more targeted approach using individualized CRRT‐specific variables and drug‐specific SC could maximize the benefit‐risk profile for each patient.

The incidence of acute kidney injury (AKI) in the intensive care unit (ICU) has increased over the past few years and ranges between 20 and 50%.^1^ Among critically ill patients, observed in‐hospital mortality incidences were over 50%.^2^ Although renal replacement therapy for endstage renal disease (ESRD) is usually managed with intermittent hemodialysis or peritoneal dialysis, these therapies can also be used in the ICU for AKI. Conventional intermittent hemodialysis can cause hypotension and cerebral edema and additionally pose difficulties in managing fluid balance. Peritoneal dialysis has limitations in cases of severe volume overload, abdominal pathology, or the need for significant solute clearance.^3^ The use of continuous renal replacement therapy (CRRT) has been progressively increasing to circumvent cardiovascular and neurological issues. CRRT is typically indicated for patients with AKI or ESRD with cardiovascular instability, septicemia, cerebral injury/edema, and rhabdomyolysis.^4^ In the ICU setting, the majority of patients are on a large number of potentially life‐saving medications, which can be impacted by clearance of these medications.^5^ Due to limited clinical data, current dosing recommendations for patients undergoing CRRT are extrapolated from patients with ESRD receiving intermittent hemodialysis as an outpatient. Because CRRT is typically provided over 24 hours for multiple days, the derived recommendations may lead to patients being under/overdosed. The need for pharmacokinetic (PK) data in this special patient population is crucial to the development of personalized dosing recommendations.^6^, ^7^, ^8^, ^9^

Status epilepticus and refractory seizures remain a common occurrence among patients in the ICU with incidence rates ranging from 19 to 34% based on continuous electroencephalogram monitoring and 76–92% in nonconvulsive patients.^10^, ^11^ Patients may require several concomitant anti‐epileptic drugs (AEDs) during their stay for the treatment and prophylaxis of diverse types of seizures. Traditional modalities of renal replacement therapy may cause worsening cerebral edema, cerebral hypoxia, increased intracranial pressure, and reduced cerebral perfusion.^12^ Therefore, CRRT could be a preferred option in critically ill patients with neurological injuries.^12^, ^13^ Patients who undergo CRRT may potentially experience refractory seizures from underexposure of therapy, whereas serious adverse effects may appear in those who are overexposed. To date, limited clinical studies assessing the impact of CRRT on AEDs have been reported and no standardized dosing recommendations have been established.^13^

Levetiracetam (LEV) is a commonly used anti‐epileptic medication in the ICU with US Food and Drug Administration (FDA) approved indications for partial onset seizures, myoclonic seizures, and primary generalized tonic‐clonic seizures in pediatric and adult patients.^14^, ^15^ Currently, guidelines also suggest that LEV can be used off‐label for seizure prophylaxis in patients with severe traumatic brain injury or subarachnoid hemorrhage for no more than 7 days.^16^ LEV exerts its antiseizure effect through modulation of synaptic vesicle 2A, a novel mechanism of action.^17^ Its PK advantages over other AEDs include rapid absorption, near 100% bioavailability when using the oral formulation, insignificant protein binding (< 10%), minimal drug‐drug interactions, and nonhepatic enzyme‐induced hydrolysis. The extent of absorption is not affected when LEV is given with food, however, studies have shown maximum concentrations (C_max_) to decrease by 20% and a delay in time to maximum concentration (T_max_) by 1.5 hours. LEV elimination is correlated with creatine clearance (CrCL) with 66% of the administered dose renally eliminated unchanged through glomerular filtration with partial tubular reabsorption. Total drug clearance is reduced by 40%, 50%, and 60% in patients with mild (CrCL: 50–80 mL/minute), moderate (CrCL: 30–50 mL/minute), and severe renal impairment (CrCL: < 30 mL/minute), respectively. LEV is also known to exhibit dose‐proportional kinetics.^15^ A reference trough concentration range of 12–46 μg/mL has been frequently reported to correlate with decreasing the occurrence of seizures in patients.^18^ However, routine therapeutic drug monitoring is not typically utilized in clinical practice.

Currently, no formal dosing recommendations exist regarding the use of LEV in patients undergoing CRRT. Given that LEV is highly water soluble, exhibits low protein binding, and has a relatively low molecular weight, one can hypothesize that LEV is highly susceptible to removal by CRRT. Current literature provides limited case reports/series from small, single center investigator initiated observational trials.^19^, ^20^, ^21^, ^22^ However, the lack of rich PK samples, effluent concentration data, flow rate settings, and filter pressures provided in the available case reports can lead to misinterpretation regarding individual dose adjustments. Case reports also include patients who are on multiple extracorporeal therapies that could potentially confound the impact of CRRT on drug PK.^22^ Continuous venovenous hemofiltration (CVVH) is one of many CRRT modalities that utilizes hydrostatic pressure to remove solutes by the process of convection. Compared with diffusion‐based modalities, such as hemodialysis, hemofiltration provides enhanced clearance of large molecular size solutes.^2^, ^4^ The objectives of this study are to characterize the PK of LEV in critically ill patients undergoing CVVH and to derive individualized dosing recommendations to optimize anti‐epileptic therapy.

## METHODS

### Study design and patient enrollment

This prospective, open label study was conducted in the medical, surgical, and neurocritical ICUs of a large academic medical center (ClinicalTrials.gov NCT03632915: PADRE‐01; completed one drug cohort but as of yet unpublished). The protocol was approved by the University of Maryland Institutional Review Board (HP‐00066222). Written informed consent was obtained from all patients enrolled either directly by the patient or their legally authorized representative. Patients were eligible for study enrollment if they were at least 18 years old, receiving CRRT for at least 24 hours, receiving LEV, and expected to survive for at least 24 hours based on the primary clinical provider’s assessment. Key exclusion criteria included: pregnancy, patient incarceration, receiving additional extracorporeal therapy, or experiencing clinically significant bleeding.

### CRRT procedures

Continuous venovenous hemofiltration therapy were performed using the PrismaFlex system (Gambro Industries, France) with the M‐150 hemofilter/dialyzer set (Baxter Healthcare, Deerfield, IL) that could be used to perform all CRRT therapies. The filter/dialyzer membrane was composed of acrylonitrile and sodium methallyl sulfonate copolymer material (AN 69 HF hollow fiber) and had an effective surface area of 1.5 m^2^. The internal fiber diameter was 240 μm and fiber wall thickness was ~ 50 μm. The blood volume of the entire extracorporeal circuit was ~ 189 mL ± 10%. Prismasate dialysate formula (Baxter Healthcare) was used as replacement and dialysate fluid. Specific fluid content formulas were based on the ordering provider’s discretion. Anticoagulation was performed, if necessary, with unfractionated heparin and was provided through the pre‐blood pump (PBP). If no anticoagulation was needed, normal saline was used as a substitute and given through the PBP at a specific flow rate. Blood flow rates, PBP fluid flow rates, and fluid therapy (net ultrafiltration flow rate; replacement fluid flow rate; dialysis flow rate; and ratio of prefilter/postfilter substitution) were prescribed at the discretion of the ordering provider and based on clinical status.

### Drug administration, sampling procedure, and bioanalytical methods

The LEV dosing regimen was selected by the clinical provider and administered either orally twice daily or as an intravenous infusion over 15 minutes twice daily. Simultaneous prefilter (red port before prefilter replacement fluid administration), postfilter (blue port before postfilter replacement fluid administration), and effluent (yellow port) samples were taken before dosing administration, after the completion of the infusion or 1 hour postoral dose, and up to six additional time points postinfusion or postoral administration. At each sampling time point, the transmembrane pressure (TMP) and filter pressure were recorded directly from the CRRT machine output. All samples were immediately placed on ice and centrifuged within 30 minutes and stored at −80°C. Total LEV plasma and effluent concentrations were determined by a validated high‐performance liquid chromatography with ultraviolet radiation detection method.^23^ All clinical samples were assayed with calibrators and quality controls and met the acceptance criteria outlined by the FDA.^24^ The limit of quantification for LEV was 2 mg/L.^23^

### Patient data collection

The following information was collected from the electronic health record: demographic data (e.g., age, sex, and race), weight, laboratory measures (serum creatinine, albumin, hematocrit, hemoglobin, International Normalized Ratio, and partial thromboplastin time), creatinine clearance, indication for CRRT therapy, length of CRRT therapy prior to study enrollment, indication for LEV therapy, number of days on LEV therapy, CRRT characteristics and flow rates, cumulative fluid removal during study, and previous 24‐hour urine output while on CRRT therapy. CRRT‐specific parameters and pressures were verified through daily progress notes, replacement fluid medication orders, and nursing hourly flowsheets. Continuous demographic data are presented as mean and SD or median and range. Categorical data are represented as counts and percentages.

### PK analysis

LEV plasma and effluent concentrations were plotted against time and individual PK parameters were calculated using noncompartmental analysis (NCA) in Pumas.jl, a Julia‐based modeling and simulation platform. Area under the curve (AUC) was calculated using the linear up log down trapezoidal method. The following formulas were used estimate the sieving coefficient (SC; Eq. 1),8 effluent flow rate (Eq. 2),25 CVVH clearance (Eq. 3),^25^, ^26^ filtration fraction (Eq. 4),27 and percentage of total drug clearance contributed by CVVH (Eq. 5)28 at each sampling time point:

(1) $$\text{SC}=\frac{C_{\text{eff}}}{C_{\text{pre}}}$$

(2) $$Q_{\text{eff}}=Q_{\text{rf}}+Q_{\text{pbp}}+Q_{\text{net}}+Q_{d}$$

(3) $${\text{CL}}_{\text{CVVH}}=SC\cdot Q_{\text{eff}}\cdot \left(\frac{Q_{b}\cdot \left(1-\text{HCT}\right)}{Q_{b}\cdot \left(1-\text{HCT}\right)+\text{PDR}\;\cdot \left(Q_{\text{rf}}\right)+Q_{\text{pbp}}}\right)$$

(4) $$\text{FF}=\frac{Q_{\text{rf}}+Q_{\text{pbp}}+Q_{\text{net}}}{Q_{b}\cdot \left(1-\text{HCT}\right)+\text{PDR}\cdot \left(Q_{\text{rf}}\right)+Q_{\text{pbp}}}$$

(5) $$\%{\text{CL}}_{\text{CVVH}}=\frac{{\text{CL}}_{\text{CVVH}}}{{\text{CL}}_{\text{NCA}}}\cdot 100$$

where SC is the sieving coefficient, $C_{\text{eff}}$ is the effluent concentration, $C_{\text{pre}}$ is the prefilter concentration, $Q_{\text{eff}}$ is the effluent flow rate (L/hour), $Q_{\text{rf}}$ is the replacement therapy flow rate (L/hour), $Q_{\text{pbp}}$ is the PBP flow rate (L/hour), $Q_{\text{net}}$ is the net fluid removal flow rate (L/hour), $Q_{d}$ is the dialysis flow rate (L/hour), ${\text{CL}}_{\text{CVVH}}$ is the CVVH clearance, $Q_{b}$ is the blood flow rate (L/hour), *HCT* is the hematocrit, *PDR* is the prefilter replacement therapy dilution ratio, FF is the filtration fraction, and ${\text{CL}}_{\text{NCA}}$ is the NCA‐derived total drug clearance (assumed to be the true total clearance in each patient). Because this study only evaluated patients undergoing hemofiltration, $Q_{d}$ was equal to zero. Exploratory graphical analysis was used to evaluate the relationship between TMP vs. SC, FF vs. SC, TMP and filter pressure over time, FF vs. $Q_{\text{rf}}$ , and ${\text{CL}}_{\text{CVVH}}$ vs. $Q_{\text{eff}}$ .

### Dose adjustment

Prospective dose adjustment calculations will be based on average individual SC, prespecified effluent flow rate, blood flow rate, and prefilter replacement therapy dilution ratio. A target area under the curve ( ${\text{AUC}}_{\text{target}}$ ) of 270 mg × hour/L (based on patients with normal renal function receiving 1,000 mg twice daily) was selected as the reference exposure of interest.^15^ The following equations were used to calculate the new dose for each patient^29^:

(6) $${\text{CL}}_{\text{tot}}={\text{CL}}_{\text{CVVH}}+{\text{CL}}_{\text{nr}}+{\text{CL}}_{\text{res}}$$

(7) $${\text{CL}}_{\text{nr}}=\frac{0.96\frac{\text{mL}}{\text{minute}}}{\text{kg}}\cdot 0.33\cdot \text{IBW}$$

(8) $${\;\text{CL}}_{\text{res}}={\text{CL}}_{\text{NCA}}-{\text{CL}}_{\text{tot}}$$

(9) $$\text{DOSE}={\text{AUC}}_{\text{target}}\cdot {\text{CL}}_{\text{tot}}$$

where ${\text{CL}}_{\text{tot}}$ is the total drug clearance (L/hour), ${\text{CL}}_{\text{nr}}$ represents the nonrenal clearance (L/hour), ${\text{CL}}_{\text{res}}$ is the residual renal clearance, and IBW is the ideal body weight in kg. Assuming ${\text{CL}}_{\text{NCA}}$ presents the true total clearance in a patient, individual ${\text{CL}}_{\text{res}}$ can be estimated using the difference between ${\text{CL}}_{\text{NCA}}$ and ${\text{CL}}_{\text{tot}}$ . Exploratory analysis to determine a quantitative relationship between ${\text{CL}}_{\text{res}}$ and 24‐hour urine output, serum creatinine, estimated glomerular filtration rate (using the Cockcroft‐Gault and the Modification of Diet in Renal Disease formula), or blood urea nitrogen was further evaluated.^30^ Current literature reports that total LEV clearance is ~ 0.96 mL/minute/kg.^15^ Nonrenal clearance pathways are composed of systemic enzymatic hydrolysis (24%) and other unknown mechanisms (< 10%).^15^ Patients with anuric ESRD demonstrated a 70% reduction in clearance as compared with patients with normal renal function.^15^ Due to the lack of any potential differences in the enzymatic activity between patients with CRRT and non‐CRRT patients and prior data in patients with anuric ESRD, nonrenal clearance was assumed to be similar to healthy patients and scaled by ideal body weight using the Devine formula.^31^

## RESULTS

### Demographic and clinical data

A total of 12 patients were recruited into the study based on the study protocol. One study patient was receiving continuous venovenous hemodialysis and was not included in the final PK analysis. Demographic and clinical data are summarized in **Table** **1**. Most patients were experiencing AKI and were in need of solute and volume management by way of CVVH therapy. Four patients received LEV therapy for seizure prophylaxis (three patients were receiving the guideline recommended dose of 1,000 mg twice daily, whereas one patient was receiving 750 mg twice daily) and seven patient received LEV for the treatment of recurrent seizures. Patient‐specific CVVH therapy characteristics are listed in **Table** **2**. Previous 24‐hour urine output indicated that a majority of patients were experiencing oliguria (24‐hour urine output < 400 mL). The average filtration fraction was ~ 27%. Patient 10006 and 10008 experienced filter clotting, and sampling was not continued. Patients 10009, 10010, 10011, and 10012 also experienced filter clotting, however, sampling was continued after blood was returned to the patient and a new filter was primed and functioning. None of the patients experienced any adverse effects attributed to LEV.

**Table 1:** Demographic and clinical characteristics

Characteristic	CVVH patients (*N* = 11)
Age, years	63.8 ± 13.2
Sex	
Male	10 (91%)
Female	1 (9%)
Weight, kg	95.7 ± 15.8
Race	
White	4 (36%)
African descent	6 (55%)
Hispanic	1 (9%)
Diagnosis	
Subarachnoid hemorrhage	3 (27%)
Intracranial hemorrhage	1 (9%)
Intraventricular hemorrhage	1 (9%)
Seizures/status epilepticus	4 (36%)
Septic shock	1 (9%)
Decompensated hepatic cirrhosis	1 (9%)
Indication for levetiracetam	
Seizure prophylaxis	4 (36%)
Treatment of seizures	7 (64%)
Indication for CRRT	
Solute and volume management	7 (64%)
Metabolic acidosis	3 (27%)
Rhabdomyolysis	1 (9%)
Renal function status	
Acute kidney injury	9 (82%)
Endstage renal disease	2 (18%)
Laboratory values	
Serum creatinine, mg/dL	2.4 ± 2.0
Albumin, g/L	2.8 ± 0.5
Hemoglobin, g/dL	8.9 ± 1.3
Hematocrit, %	26.9 ± 4.4
Prothrombin time, seconds	49.8 ± 23.3
International normalized ratio	1.3 ± 0.2
Days on CVVH therapy	2 (1–12)
Days on levetiracetam therapy	5 (2–13)
Levetiracetam dose, mg	1,000 (500–2,000)

CRRT, continuous renal replacement therapy; CVVH, continuous venovenous hemofiltration.

**Table 2:** CVVH therapy characteristics

Patient ID	Filter	$Q_{b}$ (mL/minute)	$Q_{\text{pbp}}$ (mL/hour)	$Q_{\text{rf}}$ (mL/hour)	$Q_{\text{net}}$ (mL/hour)	$Q_{\text{eff}}$ (mL/hour)	PDR (%)	Filter temperature (°C)	24‐hour urine output (mL)	FF (%)
10002	M150	250	100	2,200	0	2,300	70	44.6	332	19
10003	M150	100	100	2,000	0	2,100	70	42.8	160	35
10004	M150	150	100	3,000	0	3,100	70	43.5	175	38
10005	M150	200	70	2,150	200	2,420	70	40.9	27	23
10006	M150	200	100	1,950	100	2,250	70	42.9	462	22
10007	M150	250	100	4,000	100	4,200	50	42.8	0	30
10008	M150	200	100	2,000	0	2,100	70	42.8	613	20
10009	M150	170	100	3,300	50	3,450	70	39.1	897	32
10010	M150	200	100	4,000	0	4,100	100	41.2	90	33
10011	M150	200	100	2,400	200	2,700	70	43.1	150	27
10012	M150	300	100	3,000	50	3,150	80	44.1	1089	20

CVVH, continuous venovenous hemofiltration; FF, filtration fraction; PDR, prefilter replacement fluid dilution ratio; Q_b_, blood flow rate; Q_eff_, effluent flow rate; Q_net_, net ultrafiltration rate; Q_pbp_, pre‐blood pump rate; Q_rf_, replacement fluid flow rate.

### PK analysis

**Figure 1:**
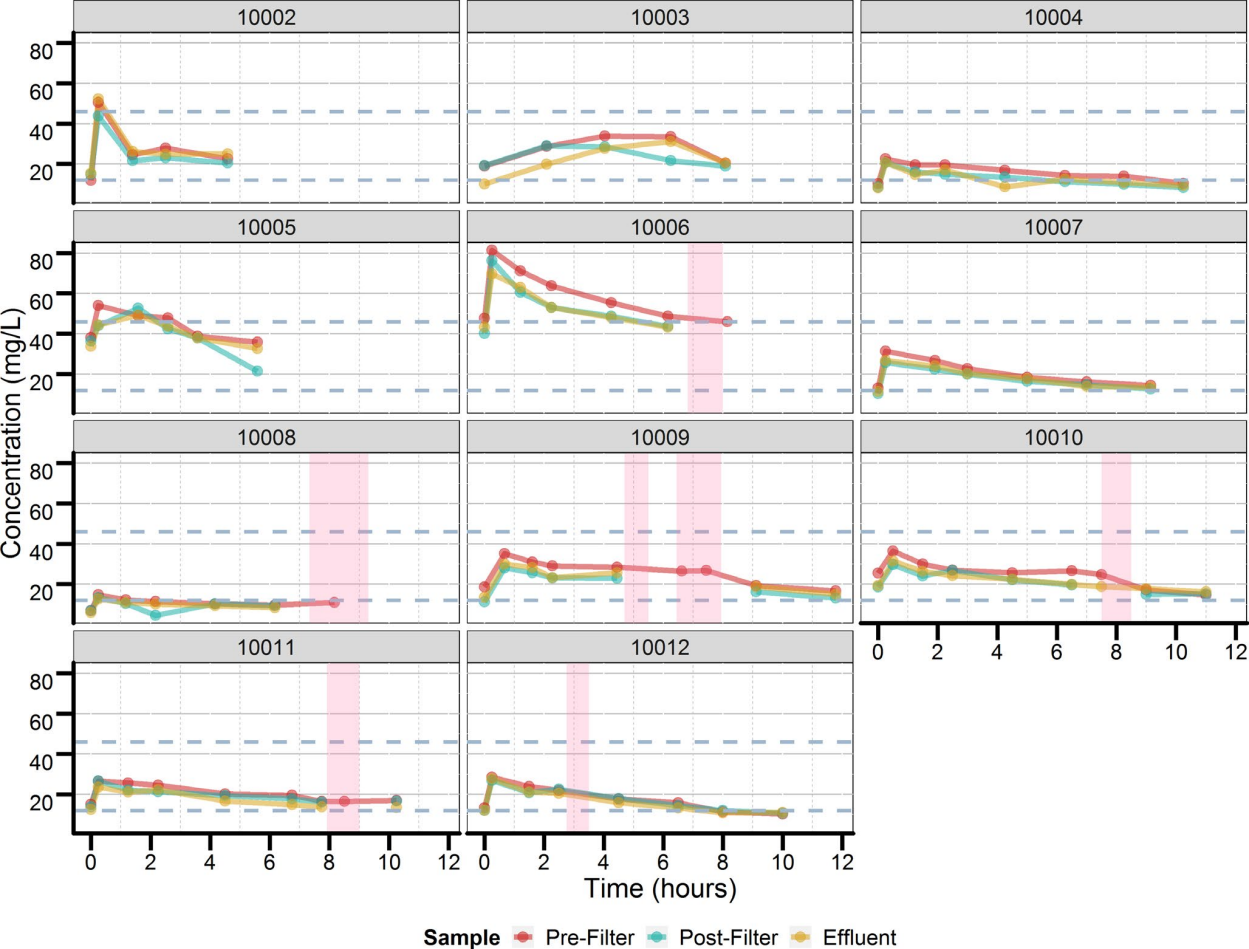
Individual prefilter, postfilter, and effluent concentration vs. time profiles. Red, blue and yellow dots and lines represent concentration data collected from the prefilter, postfilter, and effluent continuous renal replacement therapy (CRRT) sampling ports, respectively. Pink shaded region represents the range of time during the sampling period when CRRT was stopped due to filter malfunction. Gray dashed lines represent the reported therapeutic range for levetiracetam (12–46 mg/L).

**Figure 2:**
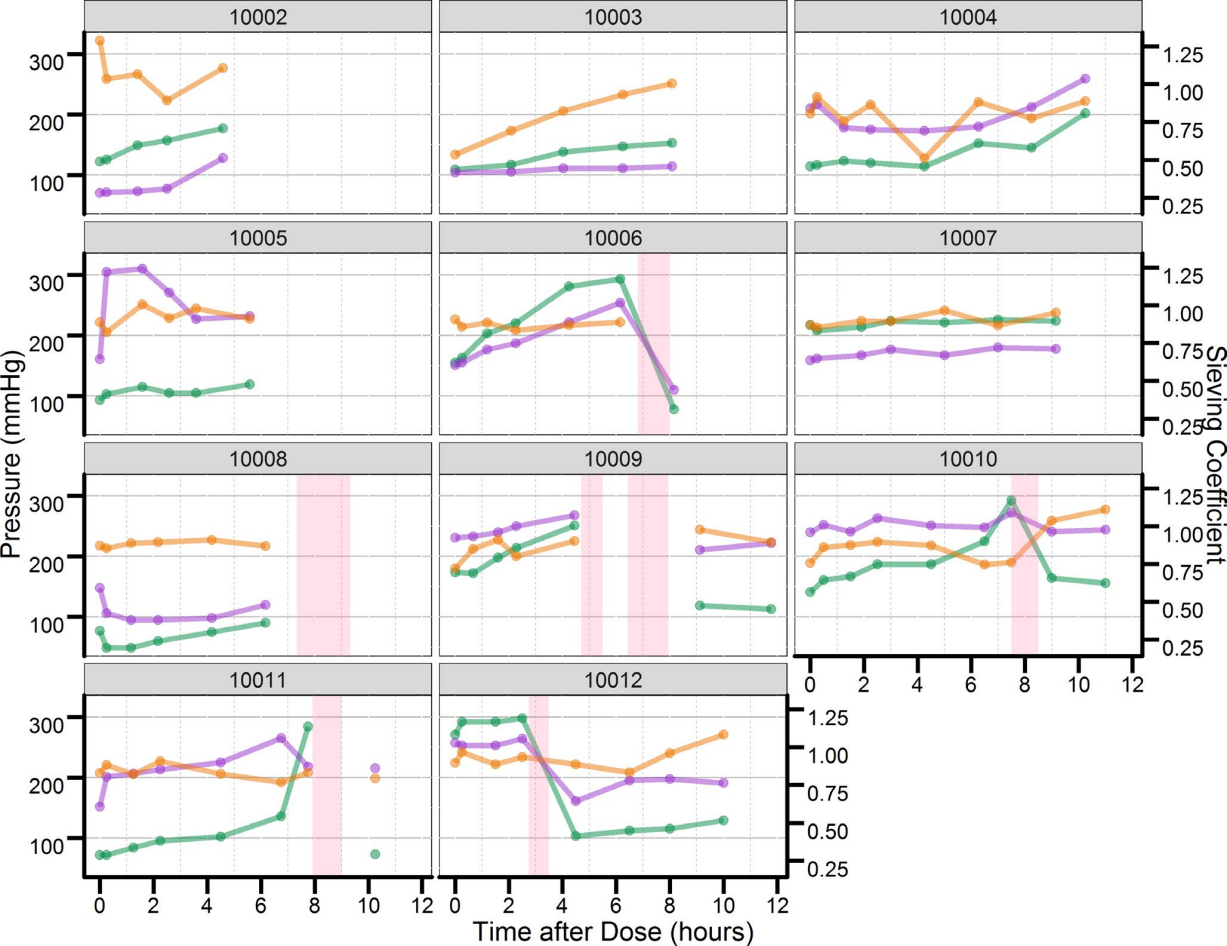
Time trend profiles for transmembrane pressure, filter pressure, and sieving coefficient. Purple, green, and orange dots and lines represent transmembrane pressure, filter pressure, and sieving coefficient, respectively. Pink shaded region represents the range of time during the sampling period when continuous renal replacement therapy was stopped due to filter malfunction.

Individual prefilter, postfilter, and effluent concentration vs. time profiles are displayed in **Figure** **1**. Analysis of concentration data during sampling times when CRRT was stopped indicates limited changes in drug concentrations, possibly indicating limited renal function and further supported by the lack of urine output in those patients. Individual PK parameters and derived CRRT parameters are provided in **Table** **3**. Patient 10003 was the only patient receiving an oral formulation. Because the patient was intubated, LEV solution was administered via the gastrostomy tube. Patient 10003 was also receiving enteral feeds, which may have attributed to a delay in the time to C_max_. This is further supported by LEV’s known food effect on decreasing C_max_ and delaying T_max_ by 1.5 hours. Using each patient’s elimination rate constant, trough concentrations (concentrations at 12 hours) were calculated and demonstrated that 6 of 11 (64%) patients exhibited concentrations outside the reported therapeutic trough range between 12 and 46 mg/L. The average volume of distribution was 51.6 L (0.73 L/kg) and is consistent with what is reported by the product label (0.7 L/kg).^15^ Total LEV clearance ranged from 2.4 to 5.48 L/hour and an average of 53% of the total clearance was attributed to CVVH therapy. The average individual SC was calculated using all available sampling time points where both prefilter and effluent concentrations were collected and ranged from 0.80 to 1.08. The overall mean and relative SD (percentage of coefficient of variation) for SC in the sample population was ~ 0.89 and 8.7%, respectively. Equation 6 was used to derive individual total LEV clearance values using average individual SC and flow rates specified in **Table** **2**. The average nonrenal clearance (CL_nr_) was 1.43 L/hour and is similar to what was previously reported (1.33 L/hour for a 70 kg patient).^15^ The average bias and precision (calculated using relative root mean squared error) of the derived total clearance value was ~ 10.6 and 21.5%. Due to changes in drug clearance over time attributed to filter clotting and cessation of CVVH therapy, a sensitivity analysis was conducted using only concentrations collected prior to returning blood from the circuit back to the patient (Table S1). Although trough concentrations at 12 hours and predicted AUC_0–12_ were numerically lower, no significant or clinically meaningful differences were observed with total LEV clearance (< 10%). **Figure** **2** displays trends in filter and transmembrane pressures over time along with changes in the SC. No time‐trend relationship between filter pressure/transmembrane pressure and SC was found. Even during periods of elevations or drops in pressures, the SC remained relatively constant. Decreases in transmembrane pressure were more prominent than changes in filter pressure after the initiation of a new filter and circuit. Exploratory analysis of CL_res_ vs. serum creatinine, EGFR, and BUN suggested no correlation and confirmed the inability to predict CL_res_ in individual patients with the collected data (**Figure** **S1**).

**Table 3:** Levetiracetam pharmacokinetic characteristics

Patient ID	Dose^c^ (mg)	*t*_1/2_ (hours)	Observed *C*_peak_ (mg/L)	Predicted *C*_trough_ (mg/L)	Predicted AUC_0–12_ (mg∙hr/L)	CL_NCA_ (L/hour)	*V*_d_ (L)	Mean SC	CL_CVVH_ (L/hour)	% CL_CVVH_	Estimated CL_nr_ (L/hour)	Estimate CL_tot_ (L/hour)	Recommended dose^d^ (mg)
10002	750	2.61	50.56	1.93	192.86	3.88	19.20	1.08	2.00	51.3	1.52	3.51	1,000
10003^a^	1,000	2.58	33.85	7.17	283.62	3.53	13.15	0.80	1.29	36.4	1.38	2.66	1,000
10004	1,000	10.35	22.56	9.94	182.63	5.48	77.99	0.80	1.97	35.9	1.52	3.49	1,500
10005	2,000	8.51	53.98	20.92	424.18	4.71	58.37	0.92	1.90	40.3	1.39	3.29	1,250
10006	1,500	10.94	81.34	34.72	625.51	2.40	38.54	0.87	1.62	67.7	1.34	2.96	750
10007	1,000	9.42	31.32	11.35	224.97	4.45	59.37	0.90	3.24	72.8	1.12	4.35	1,250
10008	500	10.66	14.86	6.35	121.48	4.11	77.18	0.88	1.68	43.5	1.52	3.21	1,000
10009^b^	1,000	8.58	35.21	16.53	302.83	3.30	43.95	0.87	2.15	65.1	1.42	3.58	1,000
10010^b^	1,000	10.06	36.45	15.31	285.49	3.50	46.87	0.88	2.45	69.8	1.65	4.10	1,000
10011	1,000	13.24	26.64	14.28	237.87	4.20	85.47	0.83	1.87	44.5	1.34	3.21	1,250
10012	1,000	6.30	28.58	7.97	193.72	5.16	47.99	0.93	2.54	49.1	1.52	4.05	1,500

%CL_CVVH_, fraction of total drug clearance attributed to continuous venovenous hemofiltration; AUC, area under the curve; CL_nr_, non‐renal clearance; CL_tot_, total clearance; *C*_peak_, pre‐filter peak concentration; *C*_trough_, prefilter trough concentration; SC, sieving coefficient; *t*_1/2_, terminal half‐life; *V*_d_, volume of distribution.

^a^ Patient 10003 received an oral solution of levetiracetam. Therefore, apparent clearance and volumes are reported.

^b^ Patients 10009 and 10010 were not samples at the end of infusion due to sampling delay.

^c^ All doses were given for a dosing interval of every 12 hours.

^d^ Recommended doses are based on matching exposures observed in healthy patients with normal renal function receiving 1,000 mg.

### Dose adjustment

Recommended doses to match exposures typically seen in patients with normal renal function receiving 1,000 mg twice daily are reported in **Table** **3**. All dose adjustments are within the recommended dose range of 1,000–3,000 mg/day. **Table** **4** provides broader dose‐adjustment regimens for an average adult patient receiving CVVH therapy at various effluent flow rates. Reference to dose adjustments listed in **Table** **4** are based on achieving exposures exhibited in patients with normal renal function receiving 500–1,500 mg every 12 hours. Different prefilter replacement fluid dilution ratios (50:50–100:0), blood flow rates (100–300 mL/minute), ideal body weight (50–70 kg), and hematocrit values (25–60%) had limited to no impact on the recommended doses. Patients with higher ideal body weights > 70 kg may require higher LEV doses. Patients with effluent flow rates > 3.5 L/hour may exhibit higher total LEV clearance may require higher doses as compared with patients with normal renal function. Given that 1,500 mg twice daily (3,000 mg/day) was the maximum dosing regimen studied across several indications, there could be a potential concern for toxicity when recommending larger doses. Although matching AUCs observed in healthy patients with normal renal function ensures similar cumulative exposures, higher doses can result in a higher C_max_ value and lead to C_max_‐dependent toxicities. To achieve a C_max_ that is less than or equal to the average C_max_ in patients with normal renal function receiving 1,500 mg twice daily, a prespecified one‐compartment PK model with a volume of distribution of 0.73 L/kg and clearance estimated using Eq. 6 was used to simulate average concentration vs. time profiles of several dosing regimens in patients with effluent flow rates ≥ 4 L/hour (**Figure** S2).^32^

**Table 4:** Dosage adjustment regimen for adult patients undergoing CVVH therapy^a^

Effluent flow rate (L/hour)	Lower exposure Dosing regimen^b^	Higher exposure Dosing regimen^b^
1	250 mg every 12 hours	750 mg every 12 hours
1.5	250 mg every 12 hours	1,000 mg every 12 hours
2	500 mg every 12 hours	1,250 mg every 12 hours
2.5	500 mg every 12 hours	1,250 mg every 12 hours
3	500 mg every 12 hours	1,500 mg every 12 hours
3.5	500 mg every 12 hours	1,500 mg every 12 hours
4	500 mg every 12 hours	1,250 mg every 8 hours
4.5	750 mg every 12 hours	1,000 mg every 6 hours
5	750 mg every 12 hours	1,000 mg every 6 hours

CVVH, continuous venovenous hemofiltration.

^a^ CVVH clearance was calculated using the following variables and range of values: hematocrit (25–60%), effluent flow rate (1–5 L/hour), blood flow rate (100–300 mL/minute), prefilter replacement therapy dilution percent (50–100%), and ideal body weight (50–70 kg).

^b^ Justification for lower and upper dose ranges are based on matching exposures (area under the curve) in patients with normal renal function receiving a dose range of 500–1500 mg every 12 hours. For effluent flow rates greater than or equal to 4 L/hour, the maximum concentrations at steady‐state for the upper dosing regimen range are maintained at or below maximum concentrations at steady‐state in patients with normal renal function receiving 1,500 mg every 12 hours.

## DISCUSSION

This proof of principle study presents data conducted from the largest clinical trial in critically ill patients undergoing CVVH and receiving LEV. Rich PK convenience sampling was conducted to fully characterize prefilter, postfilter, and effluent concentration time profiles. Individual trough concentrations suggested that only 36% of patients were within the therapeutic range. Using prior knowledge of LEV PK and principles of hemofiltration, decisions regarding individual dosing adjustments can assist in optimizing LEV therapeutics.

CVVH uses the fundamental principles of convection to provide renal replacement therapy. Convection or “solvent drag” allows for the movement of solute/drug across a semipermeable membrane. Ultrafiltration occurs when a pressure gradient between two sides of a membrane (transmembrane pressure) moves fluid with its solute content at various rates according to their SC.^33^ Drugs or solutes with molecular weight > 60 kDa (similar to molecular weight of albumin) may not be able to pass the average pore size of a membrane.^34^ To our knowledge, this study provides the first analysis of trends in transmembrane and filter pressures and evaluation of a potential relationship with SC. Membrane clogging can occur from extended use and is characterized by the formation of protein aggregation and obturation of membrane pores on the blood side of the membrane. Transmembrane pressure will consequently increase to maintain ultrafiltration; however, membrane function can become ineffective.^35^, ^36^ The lack of a relationship between transmembrane pressure and SC found in this study may imply that at the pressures observed, the impact on LEV clearance during filter clogging was minimal. Filter efficiency to extract solutes at higher pressures may also not be feasibly obtained because filters are commonly replaced when transmembrane pressures exceed 300 mmHg.

The three major determinants of solute clearance in convective therapy (CVVH) are effluent flow rates, membrane sieving properties, and dilution mode.^34^ For small solutes, the SC is approximated to the unbound fraction. Therefore, protein binding can be used as a surrogate for SC. This can be confirmed by the SC found in this study (0.89) with the unbound fraction for LEV (< 10%).^5^ Variability in individual SC was observed and could reflect the changes in protein binding due to critical illness, blood albumin content, drug displacement by other highly protein bound drugs, filter life, and the use of anticoagulation in CRRT.^37^ In this study, patients with hypoalbuminemia (< 3.5 g/dL; *N* = 9) had a similar average SC as compared with patients with normal albumin levels (0.89 vs. 0.88). A list of concomitant medications that are reported to be highly protein bound were collected for each patient. SC in patients receiving ≤ 3 (*N* = 3) and > 3 (*N* = 8) co‐administered highly protein bound drugs were also similar (0.83 vs. 0.90). No differences in SC were observed in two patients receiving regional anticoagulation of the CRRT circuit with heparin (0.90 vs. 0.88 in patients receiving anticoagulation vs. no anticoagulation)*.* Several additional case reports that discuss the PK of LEV in patients undergoing CVVH therapy were evaluated to compare the observed SC from this analysis and to estimate the bias in CL_tot_. **Table** **5** provides information on each patient’s CVVH therapy characteristics and PK summary. For the patients with effluent concentrations collected, the SC was found to be similar to what was found in this study. In the New *et al.* case, hepatic impairment played a minimal role in drug clearance and confirmed the extrapolation of CL_nr_ found in normal healthy patients. Equation 6 was used to derive CL_tot_ (assuming that SC was ~ 0.9) for each case and was similar to what was reported (percent bias ≤ 20%).

**Table 5:** CVVH therapy characteristics

Case	Filter	$Q_{b}$ (mL/minute)	$Q_{\text{eff}}$ (mL/hour)	PDR (%)	Observed SC	Observed CL_tot_ (L/hour)	Observed CL_CVVH_ (L/hour)	Observed CL_nr_ (L/hour)^a^	Observed *V*_d_ (L)	Estimated CL_tot_ (L/hour)^b^	Bias (%)^c^
Le Nobel *et al.*	AN69ST	180	3,340	43	0.89	4.07	2.98	1.10	40.5	3.98	2.2
New *et al.*	HF1400	200	2,400	50	—	3.71	—	—	60.8	4.51	−21.6
Nei *et al.*	HF1400	200	3,000	50	—	4.63	—	—	67.6	3.74	19.2
Matre *et al.*	Cartridge Express	250	2,000	—	1.03	3.68	2.25	1.43	68.3	3.11	15.5

^a^ Cases described in le Nobel *et al.*, Nei *et al.*, and New *et al.* indicated that patients were anuric. Case described by Matre *et al.* indicated that the patient produced 76 mL of urine in 12 hours.

^b^ Sieving coefficient was assumed to be 0.9 and CL_res_ was assumed to be 0 L/hour.

^c^ Bias was calculated as (100 • Observed CL_tot_ − Estimated CL_tot_/Observed CL_tot_).

The presented study has several limitations that should be acknowledged. The design of this study used a convenience PK sampling technique. Although the dosing interval was every 12 hours for all patients, several patients exhibited filter malfunctions that led to stoppage of CRRT therapy or further clinical deterioration that warranted early discontinuation of PK sampling. However, this study reflects “real‐world” situations where study results from patients receiving a variety of CVVH regimens can be generalizable. In patients where CVVH therapy was stopped, return of blood remaining in the circuit could increase plasma concentrations that could result in changes in drug exposure (AUC) and half‐life calculations. A sensitivity analysis using only concentrations prior to CVVH discontinuation suggested no difference in total drug clearance and exposure. Because patients were identified retrospectively after initiation of CVVH therapy, LEV concentrations prior to CVVH and drug clearance attributed to nonrenal pathways and residual renal function could not be accounted for. The assumption that nonrenal clearance could be extrapolated from healthy patients was based on a lack of data available describing differences in enzymatic hydrolysis between CRRT and non‐CRRT patients, similar nonrenal drug clearance in patients with anuric ESRD, and lack of dose‐adjustment needed in hepatically impaired patients.^15^, ^38^ Another limitation of this study is the application of dosing recommendations to patients experiencing clinically meaningful residual renal function (urine output > 20 mL/hour). Even though urine output can provide qualitative understanding of residual renal function, a quantitative relationship to predict CL_res_ using serum creatinine, EGFR, and BUN was not identified Although urine output is not shown to be well‐correlated with renal function, significant residual renal function is unlikely in patients with urine output of > 20 mL/hour.^30^, ^39^ In our study, only 3 of 11 patients had a total 24‐hour urine output of ≥ 480 mL. For patients undergoing any type of renal replacement therapy, quantification of residual renal function is extremely difficult.^30^ It is also important to note that creatinine and urea can also be subjected to clearance by hemofiltration, which can confound the estimation of residual renal function. In fact, the average bias calculated for the purpose of this study reflects the average fraction of residual renal function contributing to total clearance and suggests limited impact on total drug exposure. Therefore, CL_res_ was ultimately assumed to be 0 L/hour for all dosing adjustment and recommendation calculations. Last, it is unknown if the results from this study could be extrapolated to other modalities of CRRT and should be primarily applied to those patients receiving CVVH. Although this study primarily enrolled adult patients, an application to pediatrics could also be undertaken.^40^ Lower body weights in neonates and pediatric patients translate to lower blood flow rates (small catheter sizes), lower effluent flow rates (weight‐based convective CVVH regimen), and potentially lower CL_nr_ *.* Because SC, a drug‐based property, should be similar to what is observed in adults, a similar approach could be utilized to recommend individualized dosing regimens.

This study confirms a great contribution of CVVH clearance to the total LEV clearance in neurocritically ill patients. Analysis of prefilter and effluent concentrations suggests that the SC for LEV is approximated by the fraction unbound to protein and is supported by similar values observed in other case reports. Major drug clearance and dose determinants were identified to be SC and effluent flow rate. The analysis from this practice‐based study highlights dosing recommendations to achieve a target exposure range found in patients with normal renal function. In order to avoid overdosing that could lead potential toxicity and underdosing that could compromise efficacy, a more targeted approach using individualized CRRT‐specific parameters and drug‐specific SC could maximize the benefit‐risk profile for each patient.

## Funding

No funding was received for this work.

## Conflicts of Interest

The authors declared no competing interests for this work.

## Author Contributions

S.N.K., M.A., P.M., N.B., J.V.G., and M.G. wrote the manuscript. S.N.K., M.A., P.M., J.V.G., and M.G. designed the research. S.N.K., M.A., and P.M., performed the research. S.N.K., M.A., P.M, J.V.G., and M.G. analyzed the data. S.N.K., J.V.G, and M.G. contributed new reagents/analytical tools.

## Supporting information

Figure S1

Figure S2

Table S1
