# Supplementary material for: A Practice‐Based, Clinical Pharmacokinetic Study to Inform Levetiracetam Dosing in Critically Ill Patients Undergoing Continuous Venovenous Hemofiltration (PADRE‐01)
Source: Clin Transl Sci. 2020 Apr 3;13(5):950–9. doi: 10.1111/cts.12782 (PMC7485952; doi:10.1111/cts.12782)
Supplement: Supplementary file 1 — Figure S1 [file CTS-13-950-s001.docx]

**Figure S2:**

**
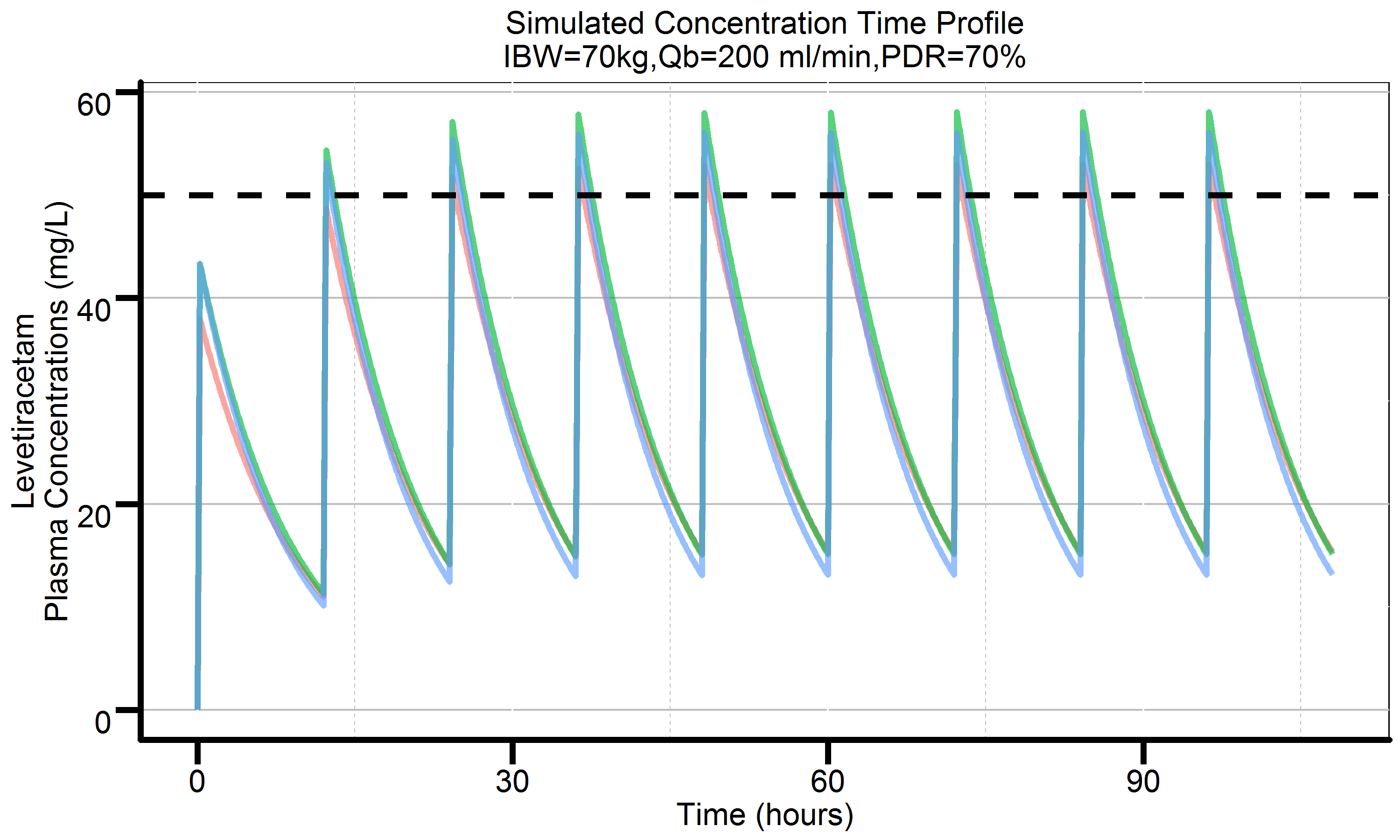
A.**

**
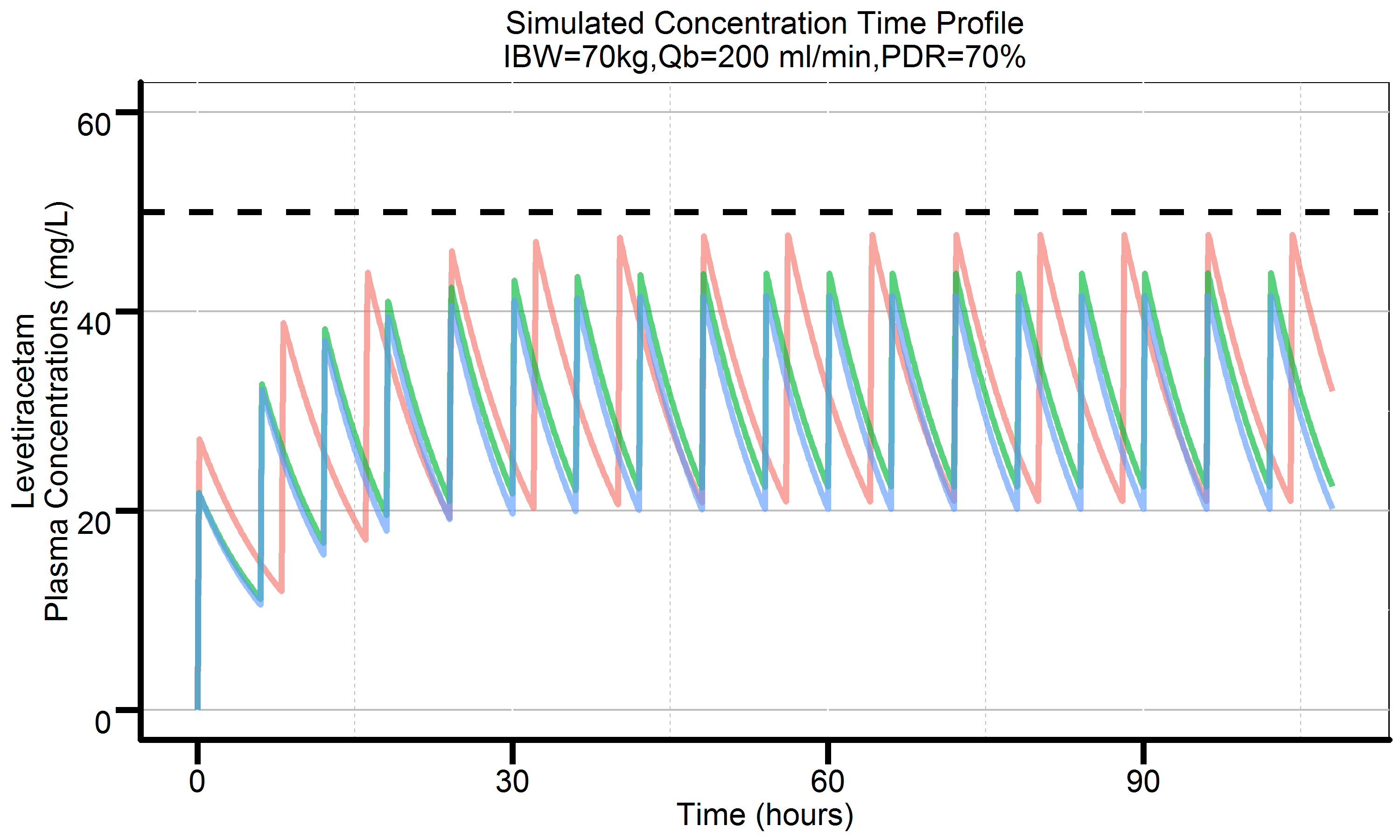
B.**

**Simulated average levetiracetam plasma concntration versus time profile.** *Black dashed line* represents the average maximum concentration at steady state in a 70 kg patient with normal renal function receiving 1500 mg every 12 hours. *Red, green, and blue lines* represent the average maximum concentration at steady state in a 70 kg patient receiving CVVH at effluent flow rates of 4,4.5, and 5 L/hrs. For panel (A), the levetiracetam dosing regimen was 1750 mg every 12 hours for flow rates of < 4.5 L/hr and 2000 mg every 12 hours for flow rates ≥ 4.5 L/hr. For panel (B), the levetiracetam dosing regimen was 1250 mg every 8 hours for flow rates of < 4.5 L/hr and 1000 mg every 6 hours for flow rates ≥ 4.5 L/hr.
