# Supplementary material for: A Practice‐Based, Clinical Pharmacokinetic Study to Inform Levetiracetam Dosing in Critically Ill Patients Undergoing Continuous Venovenous Hemofiltration (PADRE‐01)
Source: Clin Transl Sci. 2020 Apr 3;13(5):950–9. doi: 10.1111/cts.12782 (PMC7485952; doi:10.1111/cts.12782)
Supplement: Supplementary file 2 — Figure S2 [file CTS-13-950-s002.docx]

**Figure S1:**


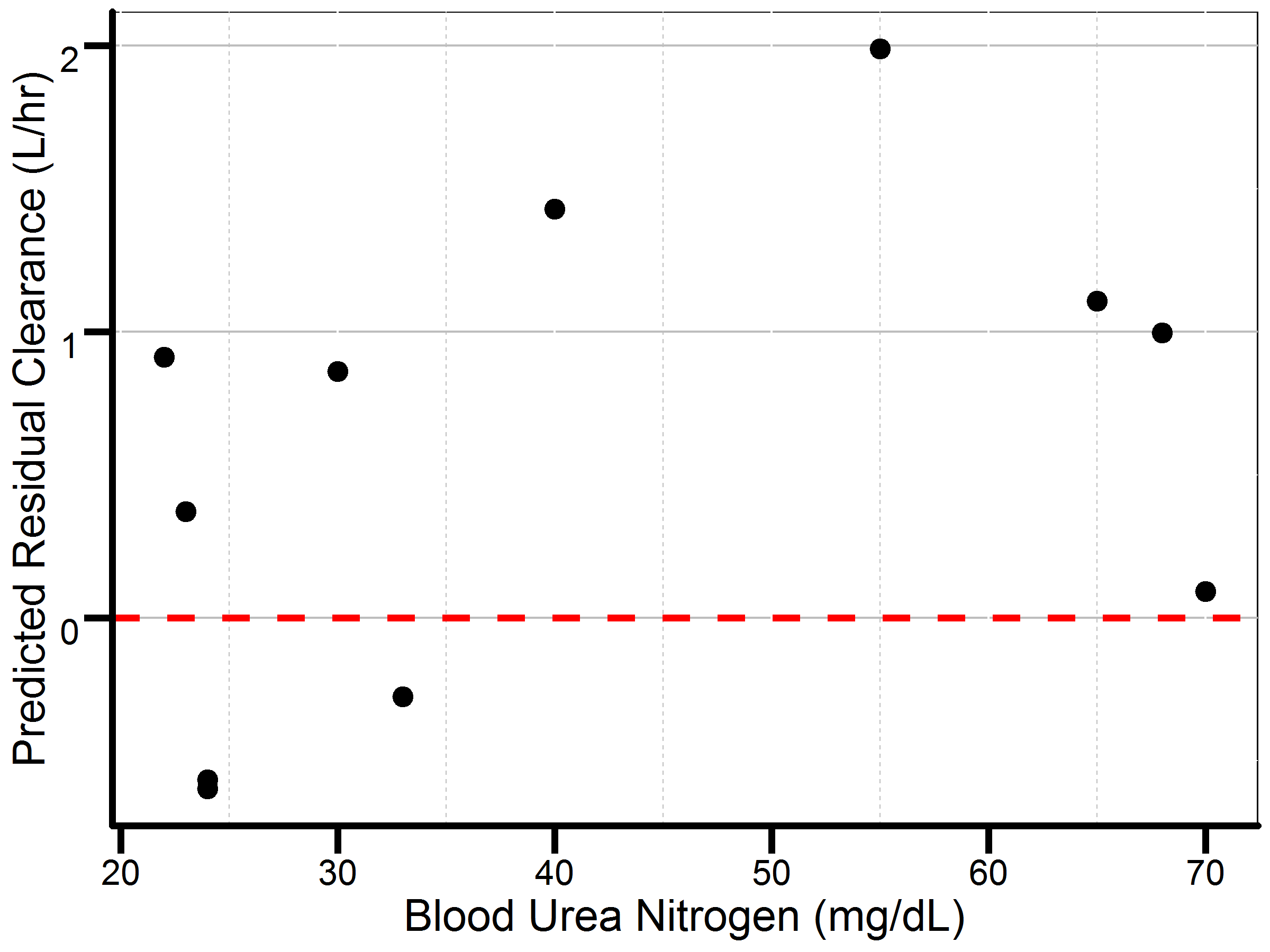

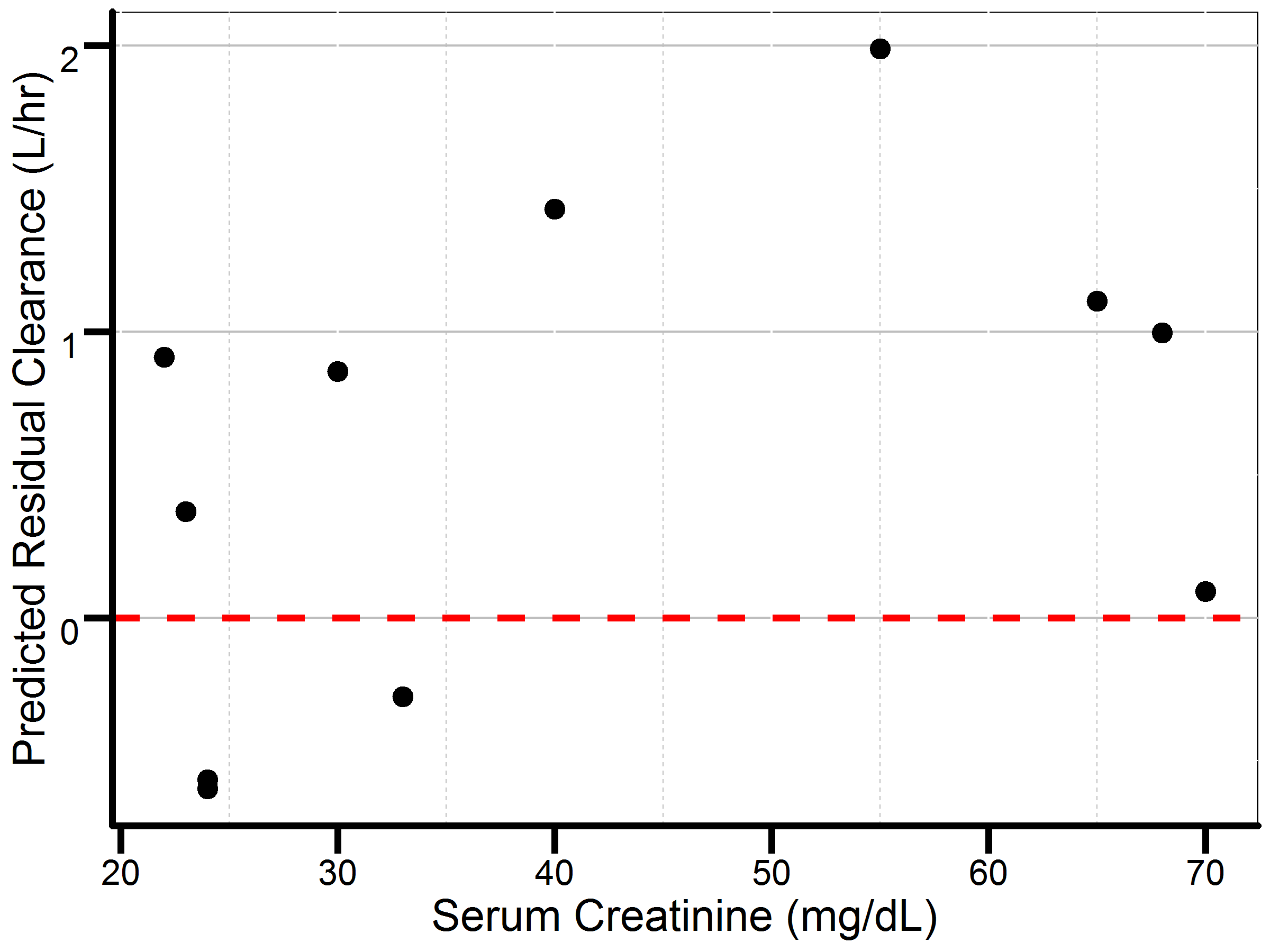
**A. B.**


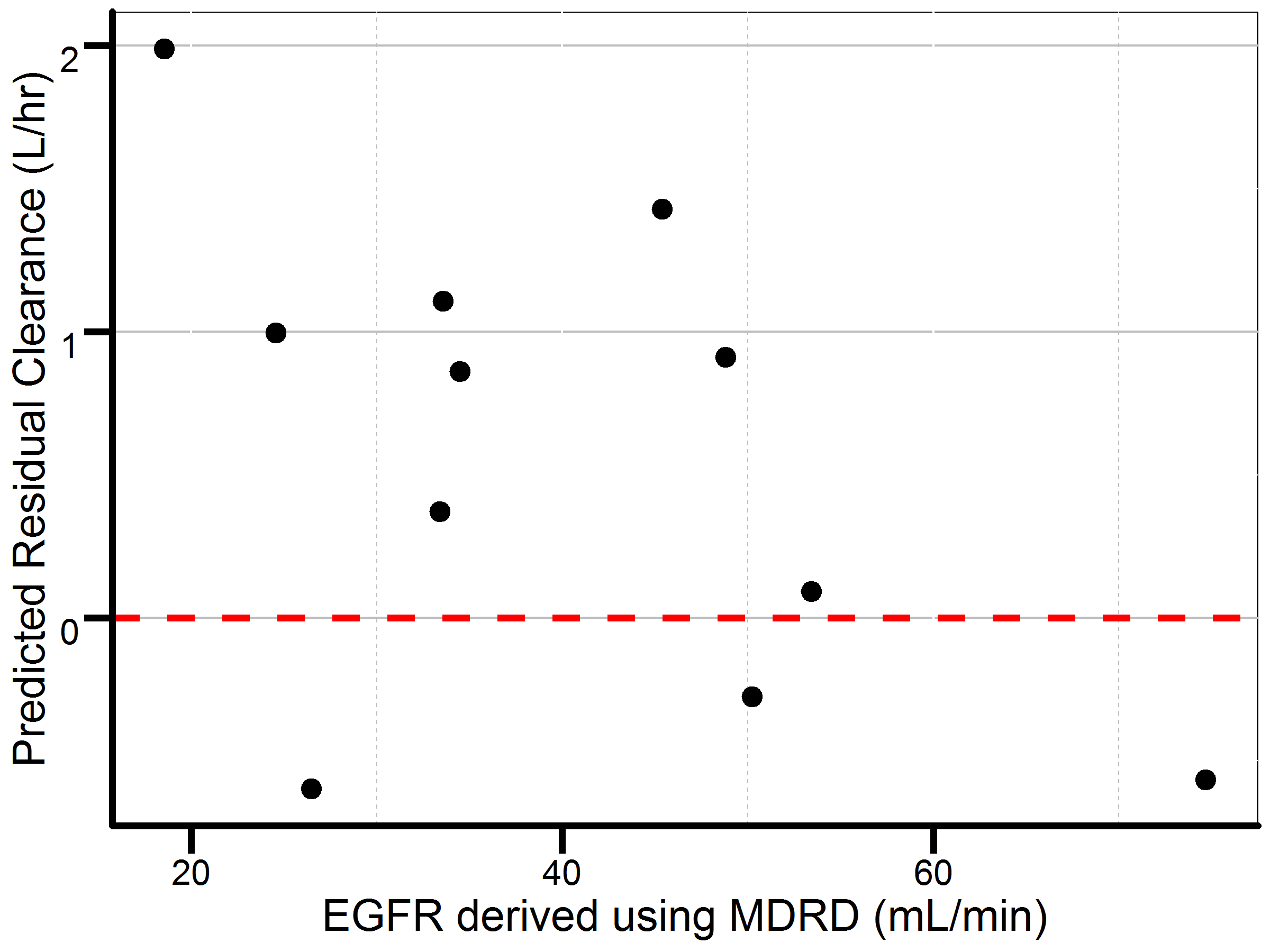

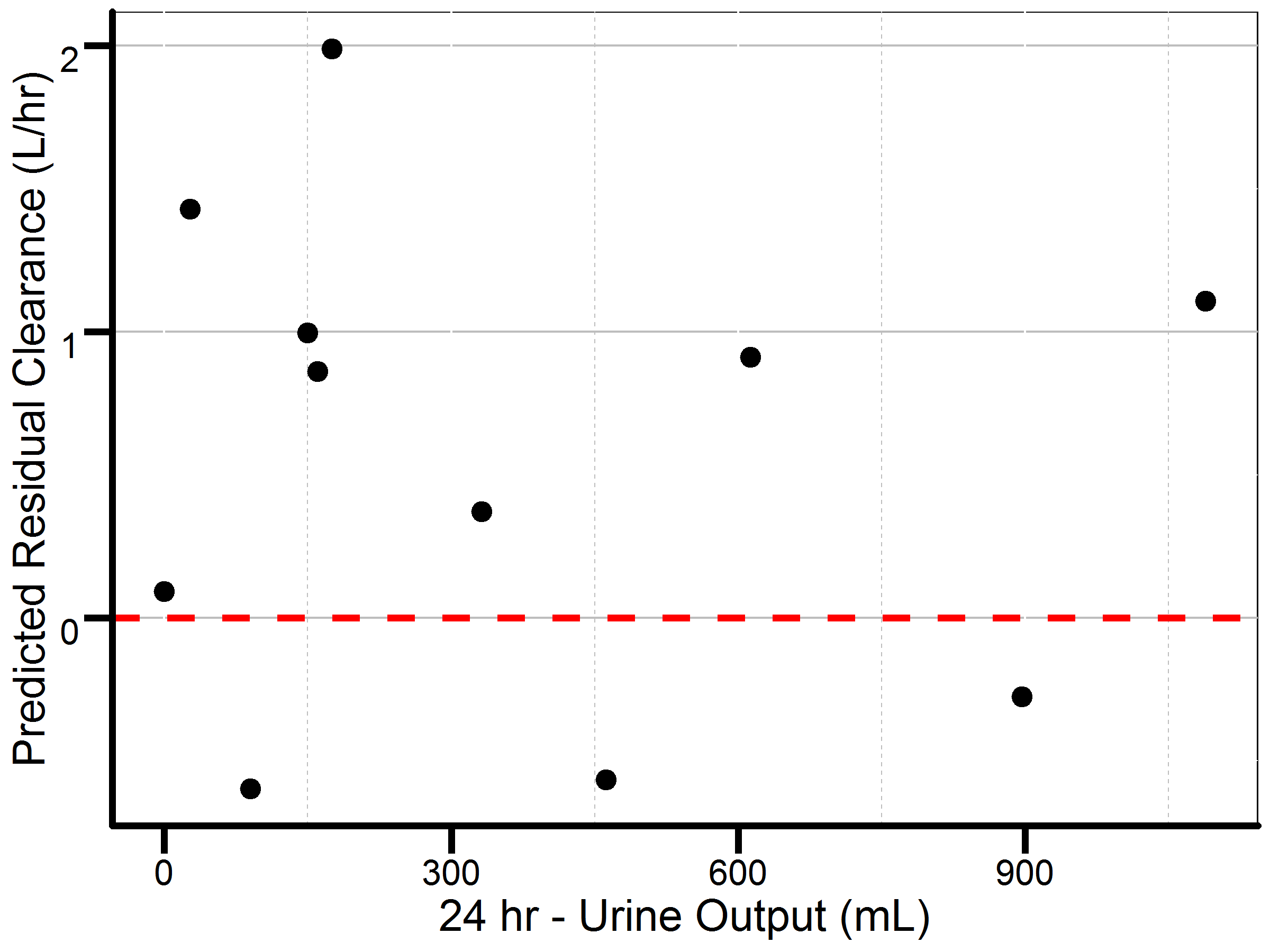
**C. D.**

**Correlation of different clinical metrics with predicted residual renal function.** Predicted residual renal function was calculated using the difference between an individual’s theoretical and NCA computed total LEV clearance. The *red line* represents a residual clearance of 0 mL/min.
