# Supplementary material for: A Practice‐Based, Clinical Pharmacokinetic Study to Inform Levetiracetam Dosing in Critically Ill Patients Undergoing Continuous Venovenous Hemofiltration (PADRE‐01)
Source: Clin Transl Sci. 2020 Apr 3;13(5):950–9. doi: 10.1111/cts.12782 (PMC7485952; doi:10.1111/cts.12782)
Supplement: Supplementary file 3 — Table S1 [file CTS-13-950-s003.docx]

**Table S1: Levetiracetam Pharmacokinetic Characteristics – Sensitivity Analysis using Concentration Data prior to Filter Clogging/Clotting**

| **Patient ID** | **Dose**^†^ **(mg)** | ***t_1/2_***  **(hours)** | **Observed *C_peak_***  **(mg/L)** | **Predicted *C_trough_***  **(mg/L)** | **Predicted *AUC_0-12_***  **(mg∙hr/L)** | ***CL_NCA_***  **(L/hr)** | ***V_d_***  **(L)** | **Mean**  ***SC*** | ***CL_CVVH_***  **(L/hr)** | **% *CL_CVVH_*** | **Estimated**  ***CL_nr_***  **(L/hr)** | **Estimate *CL_tot_***  **(L/hr)** | **Recommended Dose^†^**  **(mg)** |
| --- | --- | --- | --- | --- | --- | --- | --- | --- | --- | --- | --- | --- | --- |
| 10002 | 750 | 2.61 | 50.56 | 1.93 | 192.86 | 3.88 | 19.20 | 1.08 | 2.00 | 51.3 | 1.52 | 3.51 | 1000 |
| 10003^*^ | 1000 | 2.58 | 33.85 | 7.17 | 283.62 | 3.53 | 13.15 | 0.80 | 1.29 | 36.4 | 1.38 | 2.66 | 1000 |
| 10004 | 1000 | 10.35 | 22.56 | 9.94 | 182.63 | 5.48 | 77.99 | 0.80 | 1.97 | 35.9 | 1.52 | 3.49 | 1500 |
| 10005 | 2000 | 8.19 | 53.98 | 20.40 | 422.14 | 4.73 | 56.75 | 0.92 | 1.90 | 40.3 | 1.39 | 3.29 | 1250 |
| 10006 | 1500 | 9.26 | 81.34 | 31.20 | 606.62 | 2.47 | 32.93 | 0.87 | 1.62 | 65.6 | 1.34 | 2.96 | 750 |
| 10007 | 1000 | 9.42 | 31.32 | 11.35 | 224.97 | 4.45 | 59.37 | 0.90 | 3.24 | 72.8 | 1.12 | 4.35 | 1250 |
| 10008 | 500 | 10.66 | 14.86 | 6.35 | 114.68 | 4.36 | 69.46 | 0.88 | 1.68 | 38.5 | 1.52 | 3.20 | 1000 |
| 10009^**^ | 1000 | 5.72 | 35.21 | 8.88 | 234.49 | 4.26 | 35.76 | 0.82 | 2.04 | 47.9 | 1.42 | 3.47 | 1000 |
| 10010^**^ | 1000 | 32.63 | 36.45 | 22.92 | 318.26 | 3.14 | 150.13 | 0.83 | 2.32 | 73.9 | 1.65 | 3.97 | 1000 |
| 10011 | 1000 | 12.71 | 26.64 | 13.64 | 232.52 | 4.30 | 74.92 | 0.84 | 1.88 | 43.7 | 1.34 | 3.22 | 1250 |
| 10012 | 1000 | 6.30 | 28.58 | 7.97 | 193.72 | 5.16 | 47.99 | 0.93 | 2.53 | 49.0 | 1.52 | 4.05 | 1500 |

*t_1/2_*_:_ half-life; *C_peak_*: pre-filter peak concentration; *C_trough_*: prefilter trough concentration; *V_d_*: volume of distribution; *SC*: sieving coefficient; %*CL_CVVH_*: fraction of total drug clearance attributed to CVVH; *CL_nr_*: non-renal clearance; *CL_tot_*: total clearance

^*^Patient 10003 received an oral solution of levetiracetam. Therefore, apparent clearance and volumes are reported

^**^Patient 10009 and 10010 were not samples at the end of infusion due to sampling delay

^†^ All doses were given for a dosing interval of every 12 hours.

^††^Recommended doses are based on matching exposures observed in healthy patients with normal renal function receiving 1000mg.
